# Evolution of Superconductivity with Sr-Deficiency in Antiperovskite Oxide Sr_3−*x*_SnO

**DOI:** 10.1038/s41598-018-38403-8

**Published:** 2019-02-12

**Authors:** Mohamed Oudah, Jan Niklas Hausmann, Shinji Kitao, Atsutoshi Ikeda, Shingo Yonezawa, Makoto Seto, Yoshiteru Maeno

**Affiliations:** 1 Department of Physics, Graduate School of Science, Kyoto University, Kyoto, 606-8502 Japan; 2 Department of Chemistry, Faculty of Mathematics and Natural Sciences, Humboldt-Universität zu Berlin, Berlin, 12489 Germany; 3 Institute for Integrated Radiation and Nuclear Science, Kyoto University, Kumatori, Osaka, 590-0494 Japan

## Abstract

Bulk superconductivity was recently reported in the antiperovskite oxide Sr_3−*x*_SnO, with a possibility of hosting topological superconductivity. We investigated the evolution of superconducting properties such as the transition temperature *T*_*c*_ and the size of the diamagnetic signal, as well as normal-state electronic and crystalline properties, with varying the nominal Sr deficiency *x*_0_. Polycrystalline Sr_3−*x*_SnO was obtained up to *x*_0_ = 0:6 with a small amount of SrO impurities. The amount of impurities increases for *x*_0_ > 0.6, suggesting phase instability for high deficiency. Mössbauer spectroscopy reveals an unusual Sn^4−^ ionic state in both stoichiometric and deficient samples. By objectively analyzing superconducting diamagnetism data obtained from a large number of samples, we conclude that the optimal *x*_0_ lies in the range 0.5 < *x*_0_ < 0.6. In all superconducting samples, two superconducting phases appear concurrently that originate from Sr_3−*x*_SnO but with varying intensities. These results clarify the Sr deficiency dependence of the normal and superconducting properties of the antiperovskite oxide Sr_3−*x*_SnO will ignite future work on this class of materials.

## Introduction

Discoveries of superconductivity with high critical temperatures (*T*_*c*_’s) in the layered copper oxides^1^ and iron pnictides^2^ have opened new research fields not only on their superconductivity but also on neighboring and even wider topics such as strong correlation and multi-orbital effects in *d*-electron systems. Clarification of the composition dependence of various ordered phases and corresponding electronic properties serves as an important basis towards pioneering such novel fields. Indeed, in both copper oxides and iron pnictides, the establishment of the composition phase diagrams has been playing significant roles^3–5^. Very recently, some of the present authors reported superconductivity in the antiperovskite oxide Sr_3−*x*_SnO^6^, a new class of oxide superconductors. The superconductivity of this oxide emerges by hole doping to the parent compound Sr_3_SnO, which is unique in hosting a negative metal ion Sn^4−^ and as a consequence in exhibiting three-dimensional (3D) bulk Dirac dispersion in its electronic state^7,8^. However, it was not clear how the superconductivity emerges from the parent 3D Dirac compound as the Sr deficiency *x* is tuned and whether the negative ionic state is actually realized. In this article, we report the dependence of superconductivity on the nominal Sr deficiency *x*_0_ and reveal that the optimal *x*_0_ is located around *x*_0_ ~ 0.55–0.60. Furthermore, we provide microscopic evidence for the Sn^4−^ state in both stoichiometric and deficient Sr_3−*x*_SnO.

Antiperosvskite oxides *A*_3_*B*O (*A* = Mg, Ca, Sr, Ba, Eu, Yb and *B* = Si, Ge, Sn, Pb) have the perovskite crystal structure but with O^2−^ ions occupying the center of the octahedron formed by *A*^2+^ ions. To satisfy the charge-neutrality relation, the *B* ions take an unusual 4− oxidation state and as a consequence their *p* orbitals are almost filled^9,10^. This unusual electronic configuration can lead to interesting properties. Indeed, theoretical works on Ca_3_PbO predicted a 3D Dirac dispersion in the electronic band^7,8^, similar to recently-studied Dirac-material candidates Au2Pb^11^, Cd3As2^12,13^ and Na_3_Bi^14^. This Dirac dispersion originates from the band inversion of the nearly empty Ca-3*d* and nearly filled Pb-6*p* bands near the Γ point, as well as from the avoided hybridization between these bands due to crystal symmetry. The Dirac point is expected to have a small gap of the order of ~10 meV^7^, due to higher-order interactions originating from the spin-orbit coupling. This gapped state was later predicted to be a topological cyrstalline insulator state^15^. By changing the *A* and *B* ions, one can control the strength of the spin-orbit coupling and band mixing, and eventually tune the system from the topologically trivial insulator to the topological crystalline insulator^16^. The parent compound of this study, Sr_3_SnO, is located in the vicinity of the topological transition but still in the non-trivial regime^15^. Theoretically, it has been proposed that Sr_3−*x*_SnO can host topological superconductivity, reflecting its unusual normal-state electronic states^6,17^. More recent theoretical calculations predict various properties and deficiency effects in antiperovskite oxides, including those of Sr_3_SnO^18–20^. Furthermore, it was shown that, in Sr_2.5_SnO in different deficiency arrangements, the Fermi level still lies in bands with strong mixing between the Sr-4*d* and Sn-5*p* orbitals^21^.

Experimental works on antiperovskite oxides in the last several years were triggered by theoretical predictions^7,8,15^, and now there are reports on antiperovskite oxides in various forms, including single crystals^10,22^, polycrystals^6,23^, and thin films^24–27^. Recently, the predicted Dirac dispersion has been experimentally observed by angle-resolved photoemission spectroscopy (ARPES) using Ca_3_PbO single crystals^22^, supporting the claim from theoretical calculations. Recent ^119^Sn-NMR of nearly stoiciometric Sr_3−*x*_SnO suggests the presence of Dirac electrons in the normal state^28^.

In the initial report of superconductivity in bulk Sr_3−*x*_SnO^6^, it was proposed that hole doping due to Sr deficiency was necessary for the appearance of superconductivity. Nevertheless, quantitative analysis of the deficiency was difficult due to the uncontrolled evaporation of Sr during the synthesis. We more recently found a way to suppress the Sr evaporation^29^. In this work, we produced a large number of Sr_3−*x*_SnO samples using this method with varying the nominal deficiency *x*_0_ and examined their superconducting and normal-state properties.

## Results and Discussion

### Phase Characterization

**Figure 1:**
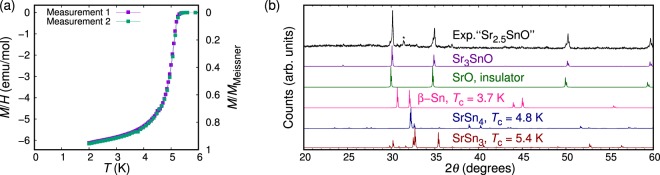
(**a**) DC magnetization measured with an applied field of 10 Oe as a function of temperature of a sample with *x*_0_ = 0.5 (Batch No. AP164). Degaussing was performed between the two measurements, both reproducibly showing strong superconducting 5-K phase, close to 100% volume fraction. Demagnetization correction was not done. After these measurements, the remnant field was checked using a reference superconductor (Pb, 99.9999% purity) and found to be below 1 Oe. (**b**) XRD pattern of the same superconductive *x*_0_ = 0.5 sample, taken after the magnetization measurements shown in (**a**), compared with expected diffraction patterns of Sr_3_SnO, SrO, *β*-Sn, SrSn_3_, and SrSn_4_^40^. *β*-Sn, SrSn_3_, and SrSn_4_ are also superconducting with *T*_c_ ’s indicated in the figure^30–33^. The vertical axis is in a linear scale. The peak marked with asterisk cannot be assigned to these impurity phases, but does not decrease in intensity even after this sample decomposes in air.

In Fig. 1(a), we present the magnetization for a superconducting sample with nominal *x*_0_ = 0.5 (chunk of 30 mg) showing *M* nearly equal to the ideal Meissner value ${M}_{{\rm{Meissner}}}=$ $-\,(1/4\pi )HV=-\,64.2\,{\rm{emu}}/{\rm{mol}}$ without the demagnetization correction, where *H* = 10 Oe is the external magnetic field and *V* = 81.0 cm^3^/mol is the molar volume. In order to allow subsequent XRD measurement, we placed the sample piece in a plastic capsule sealed with Kapton tape, all within an argon glovebox, to protect it from decomposing in air. The sample was later taken out from the capsule, and crushed into powder for XRD measurement. The measured XRD pattern for this entire piece is presented in Fig. 1(b), together with the expected diffraction patterns of Sr_3_SnO, insulating SrO, and other superconducting materials reported in the Sr-Sn-O systems^30–33^: *β*-Sn, SrSn_4_, and SrSn_3_. The XRD pattern we measured matches well with that expected for Sr_3_SnO and small shoulder peaks characteristic of SrO (cubic, *a* = 5.16 Å)^34^ can also be seen. Notice that the peak at 24.48° corresponds to the (011) peak of Sr_3_SnO^9,10^, and is not expected for SrO. This peak signifies that Sr_3−*x*_SnO is the dominant phase. The simulated XRD patterns of the other compounds do not match the measured pattern, either. From this comparison, the superconductivity with *M* close to *M*_Meissner_ certainly originates from Sr_3−*x*_SnO, providing strong confirmation for its bulk superconductivity.

**Figure 2:**
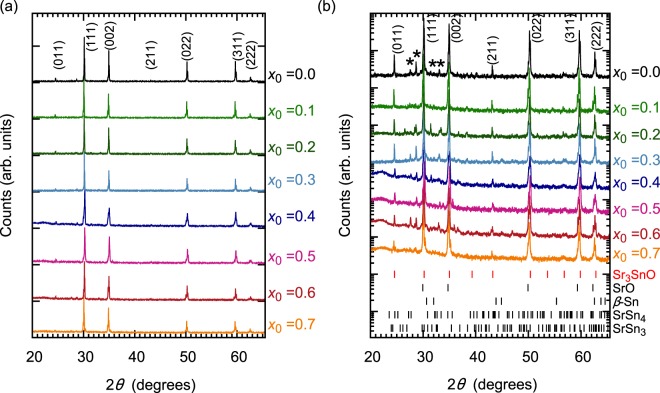
Powder XRD pattern of Sr_3−*x*_SnO samples prepared with various *x*_0_, plotted on a linear (**a**) and semi-log (**b**) scale. Each curve is shifted vertically for clarity. Minor impurity peaks from the SrO phase can be seen as the left shoulders of some peaks (such as (111), (002), (022)) of the main phase, but the shoulder was absent in the (011) peak, as expected from the crystalline symmetry of Sr_3_SnO and SrO. Additional weak peaks due to impurities (marked with asterisks) can be seen between the (011) and (002) peaks. Expected peak positions for Sr_3_SnO, SrO, *β*-Sn, SrSn_4_, and SrSn_3_ are indicated with the short vertical lines at the bottom.

In Fig. 2, we present the XRD patterns of samples prepared with *x*_0_ = 0.0–0.7. For $0.0\le {x}_{0}\le 0.6$, the dominant phase is Sr_3−*x*_SnO, as confirmed by the presence of the (011) peak. Shoulder peaks characteristic of SrO are seen on the left side of some of the main peaks, but this phase remains a minor one for ${x}_{0}\le 0.6$. For *x*_0_ = 0.7, however, the peaks of SrO become rather substantial. In addition, some peaks of additional unidentified impurity phases were observed as marked with asterisks in Fig. 2, likely originate from Sr-Sn alloys. From the XRD patterns, we evaluate the lattice constant *a* for each sample. Interestingly, *a* is found to be almost *x*_0_ independent, with *a* = 5.139 ± 0.002 Å for all *x*_0_. This fact indicates that the cubic Sr_3_SnO phase survives even with high Sr deficiency without changing the lattice constant. Such unchanging lattice parameter is similar to reports on titanium and vanadium compounds with perovskite-type structure^35,36^. The cubic phase for the perovskite titanate is preserved for the deficiency of 0.5 on the O site^35,36^, while the antiperovskite structure Sr_3−*x*_SnO survives up to the deficiency of 0.6 on the Sr site. The existence of two Sr_3−*x*_SnO phases with different deficiencies would overlap in the XRD pattern, which would explain the superconducting transitions and the Mössbauer spectra. We should comment here that other antiperovskite oxides may have various deficiency limits as observed for different perovskite oxides with different constituent elements^35^. We also comment that the deficiency in the Sr site may be accompanied by deficiency on the O site, but we expect greater deficiencies in the Sr by considering the existence of the satellite peak in Mössbauer spectra, as we will discuss in the next subsection.

**Figure 3:**
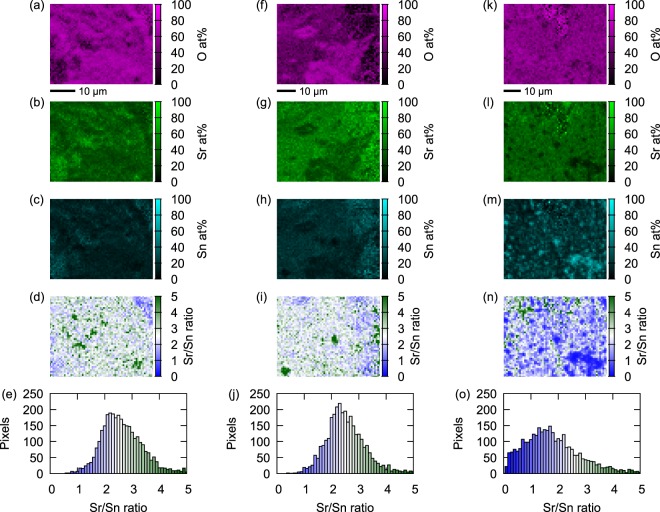
EDX results of various Sr_3−*x*_SnO samples with *x*_0_ = 0.5. The panels (a–c) respectively show mappings of the contents of O, Sr, and Sn atoms in at% value of a typical sample surface (Batch No. AP165). The ratio between Sr and Sn contents is mapped in the panel (d), and the distribution of this ratio is shown in the histogram in the panel (e). In (**d**) and (**e**), the white color corresponds to the ratio Sr/Sn = 2.5, expected for the nominal *x*_0_ value, while the green and blue colors correspond to more Sr-rich and Sn-rich ratios, respectively. The panels (f–j) present similar information but for a different region of the same sample (Batch No. AP165), and the panels (k–o) for another sample (Batch No. AP210). The sizes of the views are 41.6 × 32.5 *μ*m^2^ for (**a**–**d**), 55.4 × 43.3 *μ*m^2^ for (**f**–**i**), and 61.3 × 47.9 *μ*m^2^ for (**k**–**n**).

Representative energy dispersive x-ray spectroscopy (EDX) results for samples with *x*_0_ = 0.5 are shown in Fig. 3. We should comment that the surface of these samples was likely oxidized and decomposed during a short transfer (~1 min) from our glovebox to the EDX measurement chamber. This surface oxidization results in the high percentage of oxygen in EDX results, as seen in the panels (a), (f), and (k) of Fig. 3. However, we expect that the Sr/Sn ratio should not be drastically affected by this short exposure to air. This ratio is mapped in the panels (d), (i), and (n) of Fig. 3, where the white regions correspond to Sr/Sn = 2.5, expected from the nominal value *x*_0_ = 0.5. In these panels, we can also see some Sr-rich regions, likely originating from SrO phase in the sample. In the panel (n) of Fig. 3, we can see Sn-rich regions reflecting either an impurity formed during synthesis or decomposition on the surface during transfer to the chamber. The bottom panels show histograms of the distribution of the Sr/Sn ratio. In Fig. 3(e) and (j), the Sr/Sn ratio distribution in the investigated regions is centered around 2.5, in agreement with the Sr_2.5_SnO phase in these samples. In Fig. 3(o) the distribution is broader, but regions with the ratio close to 2.5 are still visible.

### Mössbauer Spectroscopy

**Figure 4:**
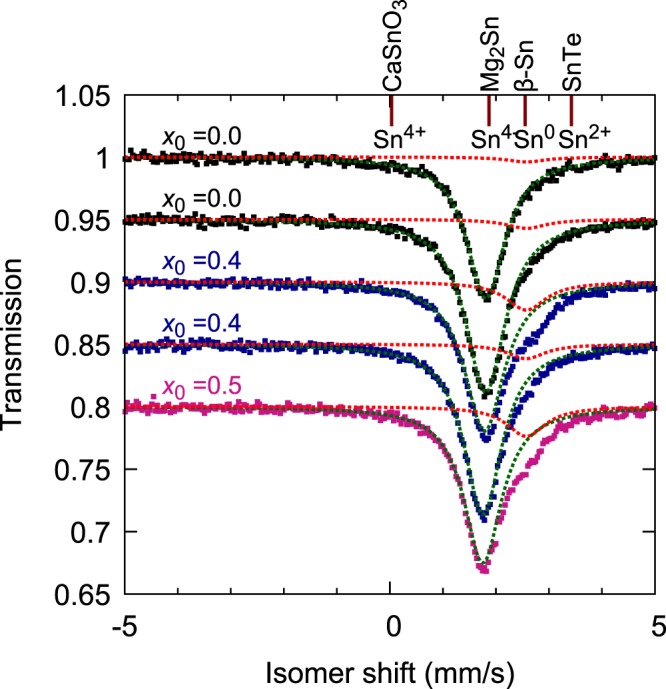
Sn Mössbauer spectra of Sr_3−*x*_SnO samples prepared with different values of *x*_0_. The origin of the isomer shift is defined as of BaSnO_3_, and isomer shifts of some reference materials^38^ with different Sn valencies are indicated with the vertical lines at the top. The dotted curves indicate Lorentzian fits for the main (green) and satellite (red) peaks. Each curve is offset vertically by 0.05 for clarity.

In order to investigate the valence of Sn ions in our samples, we performed ^119^Sn Mössbauer spectroscopy at room temperature. In Fig. 4, we present ^119^Sn- Mössbauer spectra for samples with *x*_0_ = 0.0, 0.4, and 0.5. The isomer shift, the peak position of the absorption spectra, represents the difference in the energies of the ground and excited states of the Sn nucleus of the sample, with respect to those of a reference material (with the same ionic state of Sn as the source). The source we used was CaSnO_3_ and we took the isomer shift of BaSnO_3_ as the origin, as explained in Methods.

In stoiciometric samples, the isomer shift of the main peak is about +1.8 mm/s. This shift does not match those expected for ordinary valences Sn^4+^, Sn^2+^, and Sn^0^; but is equal to that reported for Mg_2_Sn^37^, where the Sn^4−^ valence is expected based on the charge balance consideration (Mg^2+^)_2_Sn^4−^. Thus, our result provides the first microscopic support for the presence of the unusual Sn^4−^ ions with almost fully occupied Sn-5*p* orbitals. In deficient samples, we also observed the main peak at +1.80 mm/s, revealing the presence of negative Sn ions even for *x*_0_ > 0. In addition, a shoulder-like structure can be seen in the high-shift side. By fitting the overall spectrum with two Lorentzian peaks, a satellite peak centered at +2.59 mm/s is found. This peak is barely seen in the *x*_0_ = 0 samples, but we, nevertheless, fitted the *x*_0_ = 0 sample data with two Lorentzian peaks with one peak position fixed at +2.59 mm/s. The integrated peak intensity ratio of the main and satellite peaks are 100:4 for both *x*_0_ = 0 samples, 100:19 and 100:9 for the two *x*_0_ = 0.4 samples, and 100:19 for the *x*_0_ = 0.5 sample. This isomer shift of the satellite peak is close to that of *β*-Sn (+2.55 mm/s)^38^. Thus, one possible origin of this satellite peak is *β*-Sn impurity phase contained in the sample. However, this scenario is less likely considering the fact that *β*-Sn peaks in the XRD pattern is absent or quite weak in our samples (see Figs 1 and 2). Thus, presumably the satellite originates from Sn sites in Sr_3−*x*_SnO neighboring to Sr deficiency. Naively, Sn sites next to a Sr deficiency are expected to have less *p* electrons and thus to exhibit higher isomer shift due to weaker screening effect, agreeing with the experimental fact. This scenario also explains the observation that the satellite peak intensity becomes stronger for higher *x*_0_. Moreover, the existence of the satellite peak indicate that the Sn valence is clearly changed by the Sr deficiency. Thus, oxygen deficiency, which would push the Sn valency back to −4 and thus tend to avoid the Mössbauer peak change, is not significant in our samples. Notice that, even for Sr-deficient samples, a large fraction of the Sn sites is still surrounded fully by Sr without deficiencies and should exhibit Mössbauer peak at the original position. If two Sr_3−*x*_SnO phases with distinct deficiencies are in our samples, then the shoulder peak at +2.59 mm/s may originate from one of these phases.

We should comment here on the possible phase separation in the samples as indicated by the magnetization analysis (see the next subsection). Within the deficiency scenario for the origin of the satellite peak, if the sample consists of non-superconducting region with negligible deficiency and superconducting region with large deficiency of around 0.5, the former is expected to have the main peak only and the latter could show both the main and satellite peaks, with comparable intensities. Thus, the small intensity of the satellite even for the *x*_0_ = 0.5 sample agrees with the phase separation discussed later. The emergence of the satellite peak in deficient samples may be related to the observed superconductivity in Sr_3−*x*_SnO. Future investigation of the Mössbauer at low temperature in deficient superconducting samples may provide crucial information about the superconductivity in Sr_3−*x*_SnO.

### Dependence of Superconducting Properties on *x*_0_

**Figure 5:**
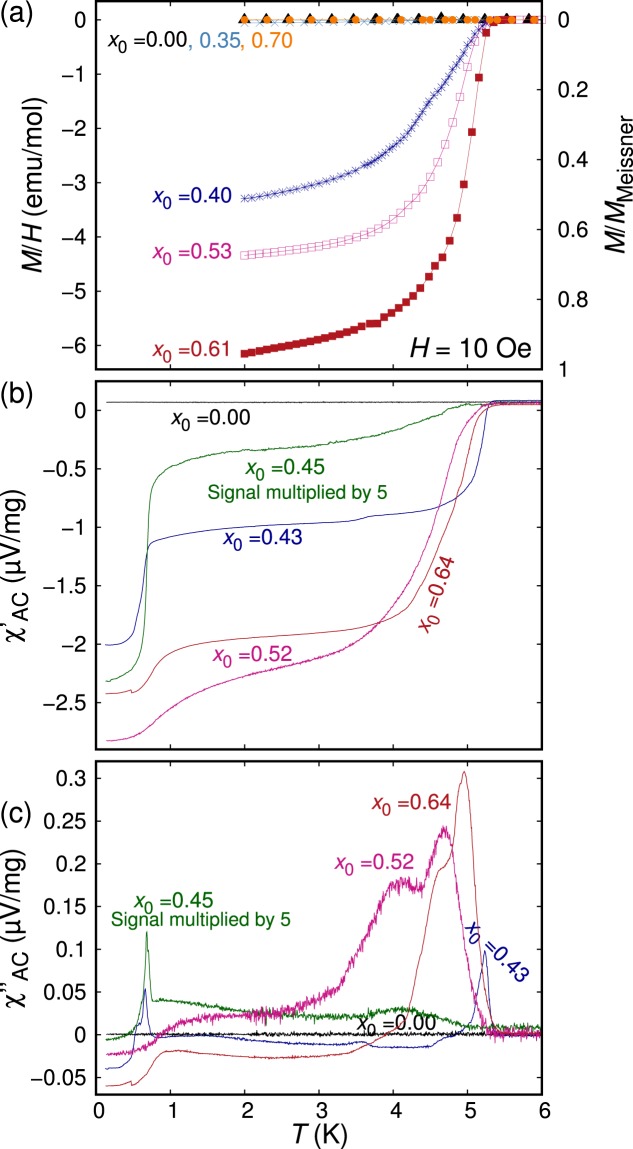
(**a**) DC magnetization as a function of temperature of Sr_3−*x*_SnO samples prepared with various values of *x*_0_. Superconductivity appears for *x*_0_ > 0.35 and becomes much weaker for *x*_0_ ≥ 0.7. (**b**) Real and (**c**) imaginary parts of AC susceptibility, *χ*_AC_, normalized by the sample mass, plotted as functions of temperature. Two superconducting transitions at 5 K and 1 K appear for superconducting samples.

Figure 5(a) represents the temperature dependence of DC magnetization down to 1.8 K of representative Sr_3−*x*_SnO samples prepared with various values of *x*_0_. Superconductivity appears for some samples with 0.35 < *x*_0_ < 0.70. The onset *T*_c_ is observed to be commonly 5 K for such superconductive samples, but the ratio *M*/*M*_Meissner_ at 2 K varies a lot. These facts suggest some inhomogeneity in the samples: our samples consist of regions with different deficiency, non-superconducting region with small *x* and superconductive region with large *x*. We emphasize again that *M*/*M*_Meissner_ close to 1 in zero-field-cooling (ZFC) measurements observed in some samples provides strong evidence for the bulk superconductivity of Sr_3−*x*_SnO, considering the sample purity demonstrated by XRD.

**Figure 6:**
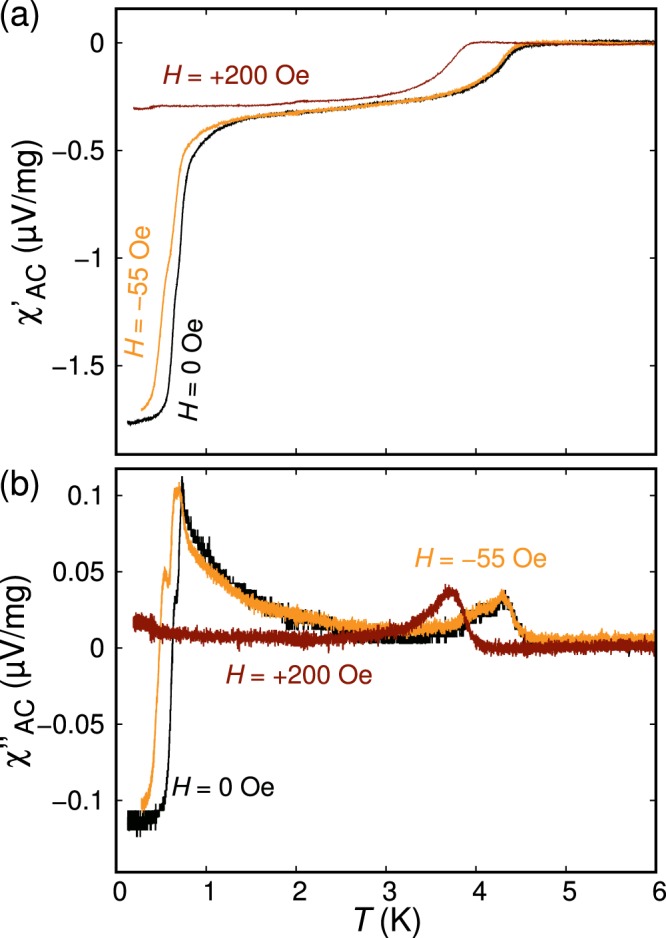
Temperature dependence of (**a**) real and (**b**) imaginary parts of AC susceptibility, *χ*_AC_, normalized by the sample mass, measured under various magnetic fields to emphasize the effect of magnetic field on the 1 K superconducting phase. The measurements were performed for a sample with *x*_0_ = 0.3. The magnetic field values indicated in the figure is evaluated considering the estimated remnant field of 125 Oe after the adiabatic demagnetization refrigeration process. Because this sample was made with a different Sr (Furuuchi, 99.9%), results of this sample are not included in other figures.

In Fig. 5(b) and (c), the real and imaginary parts of the *χ*_AC_ signal normalized by the sample mass are shown for representative samples. Interestingly, another superconducting transition appears at ~1 K for all superconducting samples. The magnitude of the superconducting signals of these two superconducting phases varies depending on the sample. In the examples shown in Fig. 5(b) and (c), the *x*_0_ = 0.52 sample exhibits a stronger transition at 5 K, while in the *x*_0_ = 0.43 sample the 5-K and 1-K transitions have similar magnitudes. This fact indicates that two transitions originate from different parts of a sample, presumably with slightly different Sr contents. The magnetic field effect on the 1-K phase was investigated as plotted in Fig. 6, where *χ*_AC_(*T*) curves under different magnetic fields are shown. The 1-K superconducting phase completely disappears at 200 Oe, indicating that the upper critical field of the 1-K phase is less than this value.

**Figure 7:**
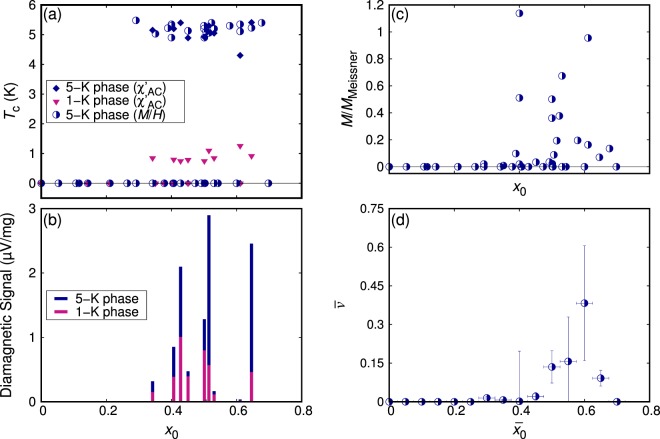
(**a**) *T*_*c*_ as a function of *x*_0_ based on the DC magnetization (down to 1.8 K) and the AC susceptibility (down to 0.1 K). (**b**) Mass-normalized diamagnetic signal *χ*_AC_ of the 5-K and 1-K phases. (**c**) *M*/*M*_Meissner_ evaluated using DC magnetization data at 1.8 K, without demagnetization correction. (**d**) Mass-weighted average of *M*/*M*_Meissner_, $\bar{v}$, taken over 0.05 intervals of *x*_0_ indicated by horizontal bars. The vertical error bars indicate the weighted standard errors.

The *x*_0_ dependence of superconducting properties, namely *T*_c_ and the size of the diamagnetic signal, evaluated based on the DC magnetization and *χ*_AC_ measurements are summarized in Fig. 7. Superconductivity with *T*_c_ of about 5 K and 1 K appears in the range 0.35 ≤ *x*_0_ ≤ 0.65, with almost no change of *T*_c_, as shown in Fig. 7(a). Figure 7(c) shows the ratio *M*/*M*_Meissner_ of 45 samples, corresponding to the volume fraction without demagnetization correction, of the 5-K phase superconductivity calculated using the DC magnetization of the ZFC process at 1 Oe and 1.8 K. The ratio is strongly sample-dependent even among samples with similar *x*_0_ values. Nevertheless, there is a tendency that strongly superconducting samples are more likely to be found around *x*_0_ ~ 0.5. To clarify this tendency more objectively and more quantitatively, we evaluate the mass-weighted average of the *M*/*M*_Meissner_ ratio, ${\bar{v}}_{{\bar{x}}_{0}}$, of the range ${\bar{x}}_{0}-\,0.025 < {x}_{0} < {\bar{x}}_{0}+0.025$ as

1 $${\bar{v}}_{{\bar{x}}_{0}}=\frac{\sum _{i}{m}_{i}{v}_{i}}{\sum _{i}{m}_{i}}$$

where *m*_*i*_ and *v*_*i*_ are the mass and *M*/*M*_Meissner_ of the *i*-th sample and summation over *i* is taken for samples whose *x*_0_ value is in the range mentioned above. The results are presented in Fig. 7(d). Here, a dome-like-shaped peak appears centered at ${\bar{x}}_{0}=0.55\,\sim \,0.60$; hinting at a specific phase favorable for superconductivity.

The normalized *χ*_AC_ diamagnetic signal of the 5-K and 1-K phases are summarized in Fig. 7(b). Here, the changes in the signal from 6 K to 2 K and from 2 K to 0.1 K are chosen to represent the diamagnetic signal of the 5-K and 1-K phases, respectively. Some samples are dominated by the 1-K phase, while others have a stronger contribution from the 5-K phase. Nevertheless, we again observe a tendency that high signals are found for samples with *x*_0_ ~ 0.5. We comment here that the *M*/*M*_Meissner_ presented in Fig. 7(c) may be an underestimate due to dominance of the 1-K phase in some of these samples, such as the *x*_0_ = 0.45 sample presented in Fig. 5(b).

The results presented in Fig. 7 indicate that the samples contain three different regions with different deficiencies: non-superconducting parts and two parts exhibiting superconductivity at 5 K and 1 K. Thus, the change in the nominal deficiency *x*_0_ results in changes in the relative volume fractions of these phases, but not in the change of the actual deficiency in each of these phases. Let us define here the actual *x* values for the non-superconducting part as *x*_n_, that for the 5-K superconducting region as *x*_5K_ and that for the 1-K superconducting region as *x*_1K_. Because samples with *x*_0_ < 0.35 do not exhibit superconductivity, *x*_n_ is probably close to 0. From the analysis in Fig. 7(d), *x*_5K_ probably lies in the range 0.55–0.60. Since all superconducting sample exhibit both the 5-K and 1-K superconductivity, *x*_1K_ must be close to *x*_5K_. Comparing Fig. 7(b) and (d), the peak in the 1-K superconductivity volume is located at the lower deficiency side. Thus, *x*_1K_ is expected to be slightly smaller than *x*_5K_. It is also possible that these two superconductive phases differ in the oxygen stoichiometry. Concurrency of the two superconducting phases with *x*_5K_ and *x*_1K_ may result from phase stability feature near the reaction temperature: there may be two thermodynamically stable phases with *x*_5K_ and *x*_1K_ and actual samples exhibit phase separation to these two phases during the growth. Control of such phase separation is not yet achieved but should be tried in future. In addition, carrier doping by methods other than deficiency, such as substitution of Sr with K or Na, will provide hints toward clarifying this issue.

## Conclusion

In summary, we have reported comprehensive bulk and microscopic investigation of Sr_3−*x*_SnO samples with the nominal Sr deficiency *x*_0_ varying from 0.0 to 0.7. We provided evidence for the unusual Sn^4−^ state with the filled 5*p* orbital in both stoichiometric and deficient samples. We have demonstrated that superconductivity appears for samples with 0.35 < *x*_0_ < 0.70. All superconducting samples exhibit two superconducting transitions, at about 5 K and 1 K. The present findings, clarifying the composition necessary for the appearance of superconductivity in Sr_3−*x*_SnO, serve as important bases toward investigation of the proposed topological superconductivity in this system^6,17^. Producing superconducting Sr_3−*x*_SnO single crystals or thin-films can be a next important step to the goal.

## Methods

Bulk polycrystalline Sr_3−*x*_SnO samples were prepared by heating mixtures of the starting materials Sr (Aldrich, 99.99%) and SnO (Furuuchi, 99.9%) in varying ratios Sr:SnO = (3−*x*_0_):1 to control the amount of Sr deficiency. Reaction was carried out at 825 °C in an alumina crucible inside a quartz tube sealed with 0.3 atm (at room temperature) of argon. Sr (Furuuchi, 99.9%) was used only for the sample shown in Fig. 6. Details of the synthesis are described in ref.^29^. Throughout this article, *x*_0_ refers to the nominal value. Powder X-ray diffraction (XRD) patterns were collected for various samples using a commercial diffractometer (Bruker AXS, D8 Advance) utilizing the CuK_*α*_ radiation. The samples were placed on a glass stage inside a glovebox and covered with a 12-*μ*m-thick polyimide film (DuPont, Kapton), which was attached to the sample stage with vacuum grease (Dow Corning Toray). With this setup, we minimized contact of the samples with air, and we confirmed that the sample degradation is negligible within typical measurement time of 200 min. The lattice constant was estimated using WPPD method using the software TOPAS. The chemical composition at the sample surface was characterized using an energy dispersive X-ray spectroscopy (EDX) system, a scanning electron microscope (Keyence, VE-9800) equipped with an X-ray detector (AMETEK, Element K). Mössbauer spectra were collected using Ca^119*m*^SnO_3_ *γ*-ray source and the origin of the isomer shift was chosen to be that of BaSnO_3_. The isomer shift is closely related to the local electronic density at the nucleus position. Thus, the isomer shift is most sensitive to the number of *s* electrons, which has a large wavefunction weight at the nuclear position, whereas *p* and *d* electrons lead to opposite weaker shift compared to *s* electrons via the screening effect. DC magnetization was measured using a commercial superconducting quantum interference device (SQUID) magnetometer (Quantum Design, MPMS), while *χ*_AC_ was measured using a miniature susceptometer^39^, which was installed in a commercial cryostat (Quantum Design, PPMS) with an adiabatic demagnetization refrigerator (ADR) option.
